# Dog Walking and Physical Activity in the United States

**Published:** 2006-03-15

**Authors:** Sandra A Ham, Jacqueline Epping

**Affiliations:** Physical Activity and Health Branch, Division of Nutrition and Physical Activity, Centers for Disease Control and Prevention; Physical Activity and Health Branch, Division of Nutrition and Physical Activity, Centers for Disease Control and Prevention, Atlanta, Ga

## Abstract

**Introduction:**

Dog walking is a purposeful physical activity that may have health benefits for humans and canines. A descriptive epidemiology of the contribution of dog walking to physically active lifestyles among dog walkers in the United States has not been previously reported.

**Methods:**

Data on youth and adults who reported walking for pet care trips (N = 1282) on the National Household Travel Survey 2001 were analyzed for number of trips, proportion walking a dog for at least 10 minutes on one trip, and accumulation of 30 minutes or more in 1 day of walks lasting at least 10 minutes.

**Results:**

In 1 day, 58.9% of dog walkers took two or more walks, 80.2% took at least one walk of 10 minutes or more, and 42.3% accumulated 30 minutes or more from walks lasting at least 10 minutes each. There were no significant differences by sex, family income, or categories of urbanization.

**Conclusion:**

Walking a dog may contribute to a physically active lifestyle and should be promoted as a strategy that fits within the framework set forth by the Task Force on Community Preventive Services for Physical Activity.

## Introduction

Regular physical activity is important for preventing obesity and other chronic diseases (e.g., coronary heart disease, diabetes, breast cancer, colon cancer), disabling conditions (e.g., osteoporosis, arthritis) and risk factors for chronic disease (e.g., hypertension, high cholesterol) (1). Despite these substantial health benefits, fewer than one half of adults engage in recommended levels of physical activity (2), and nearly 25% of adults do not participate in any leisure-time physical activity (3). In 2003, 45% of youth in grades 9 through 12 had either no participation at all or insufficient participation in moderate and vigorous physical activities (4). Recommendations for adults and adolescents are to accumulate 30 minutes or more of moderate-intensity physical activity on most, preferably all, days of the week (5). Moderate physical activity is performed at an intensity equivalent to brisk walking for most healthy adults. Walking is one of the most popular leisure-time physical activities among adults (6) and has been associated with long-term adherence to regular physical activity (7).

Walking a dog could help a large proportion of the U.S. population to increase their physical activity as well as that of their dogs. There are approximately 65 million dogs in U.S. households (8); 39% of U.S. households include at least one dog, and 35% have two or more. An estimated 25% to 40% of dogs in the United States are overweight or obese (9). Inactivity has been shown to be a significant risk factor for obesity in studies of dogs and cats, and both sedentary pet behavior and owner lifestyle may contribute to the development of canine and feline obesity (9). The Humane Society of the United States recommends twice daily walking for dogs' health and fitness (10).

Dog walking as a method for increasing human physical activity has not been extensively studied. In Australia, people who walked their dogs walked 18 minutes per week more and were more likely to meet physical activity recommendations of 150 minutes per week than people who did not own dogs (11). A study in the United Kingdom found that new dog owners accumulated significantly more walking than either new cat owners or adults without pets (12). Finally, an intervention study in the United States found that dog-related activity accounted for about two-thirds of total physical activity in overweight and obese dog owners (13).

A descriptive epidemiology of dog walking among people who walk dogs in the United States has not been previously reported. This study uses data from a national transportation survey to estimate the contribution of dog walking to physical activity among people in the United States who walk dogs.

## Methods

### Data collection

The 2001 National Household Travel Survey (NHTS), a cross-sectional survey of personal transportation by the civilian, noninstitutionalized population in the United States, was conducted by the U.S. Department of Transportation. From March 2001 to May 2002, trips made by any means of transportation were reported for 160,758 people, from infants through adults aged 88 years, using 24-hour travel diaries (14). Households were selected using random-digit dialing. All household members were asked in an initial household interview to complete travel diaries for a randomly assigned day and to report back in a follow-up telephone interview. Proxy interviews were used for youths younger than 16 years. The response rate was computed for the households in which at least 50% of adults completed both interviews; only data from this sample were released for public use and were analyzed for the present study. The overall response rate for completing the two interviews and reporting the diary data was 29.4% for individuals in these households; 91.4% of individuals in these households provided complete diaries (14). Modes of travel, duration of trips, and purposes for trips were collected using open-ended questions; responses were categorized by interviewers. Walking trips for the purpose of "pet care: walk the dog/vet visits" were considered dog walks. A dog walk was considered a bout of physical activity if it lasted at least 10 minutes. Durations for all dog walks were summed for each respondent. Individuals were further classified by whether or not they walked a dog for 30 minutes or more in 1 day accumulated in walks of at least 10 minutes each. Trips were classified by five urbanization categories (urban, second city, suburban, small town, and rural) based on the classification of the census block group in which the respondent's household was located (14). Second cities are secondary population centers in urbanized areas. For this study, we analyzed 2398 dog-walking trips made by 1282 individuals.

### Statistical analysis

Estimates for the proportion of U.S. dog walkers in this sample who walked a dog at least twice in 1 day and for the proportion who walked a dog at least once in 1 day (for 10 minutes or more) are reported by sex, age, family income, region, and urbanicity, as are estimates for the proportion of dog walkers who accumulated 30 minutes or more of dog walking in 1 day from walks of at least 10 minutes each. A logistic regression model for 30 minutes or more of dog walking was adjusted for sex, age, family income, geographic region, and category of urbanization. Adjusted odds ratios (ORs) and 95% confidence intervals (CIs) are reported. Data were weighted to adjust for nonresponse and selection bias and to represent all daily travel made by the U.S. population in 2001. Adjustment factors were applied to households, and then weights were applied to participants using U.S. Census 2001 population estimates (e.g., age, sex, race and ethnicity, day of week, month, census region, household size, size of metropolitan area). SUDAAN version 8.0 (RTI International, Research Triangle Park, NC) was used for statistical analyses.

## Results

Most (89.4%) of the individuals in the sample who reported dog walking (N = 1282) were adults, 55.4% were aged 45 years or older, and 58.1% had annual family incomes of more than $50,000 (Table 1). Seventy percent resided in the Northeast or Midwest. Among individuals in the sample, 10.8% lived in urban areas, 49.2% in second city or suburban areas, and 40.0% lived in less densely populated towns and rural areas.

Among individuals in the sample who walked their dogs, an estimated 58.9% (95% CI, 53.3%–64.4%) engaged in two or more walks in 1 day, and an estimated 80.2% (95% CI, 75.3%–84.4%) engaged in one or more walks of at least 10 minutes in 1 day (Table 2). An estimated 55.6% (95% CI, 48.5%–62.4%) of females and 63.4% (95% CI, 56.7–69.7) of males walked dogs two or more times during the day. An estimated 42.3% (95% CI, 37.3%–47.5%) of dog walkers accumulated at least 30 minutes of walking from walks of at least 10 minutes each (Table 3). When adults aged 45 years or older were used as the referent group, the OR for youths walking the dog for 30 minutes or more was 0.37 (95% CI, 0.17–0.77) (Table 4). Using the Midwest as the referent group, the OR for accumulating 30 minutes or more of dog walking in the Northeast was 2.03 (95% CI, 1.08–3.81). No other significant differences were seen by age, sex, family income, region, or urbanicity.

## Discussion

This study suggests that nearly half of adults in this sample who walked a dog accumulated 30 minutes or more of walking in bouts of at least 10 minutes each in 1 day and that neither sex nor income level made a significant difference in whether this amount of dog walking was accomplished. Although for some people the pace of dog walking may be slower than is recommended for physical activity, dog walking may contribute to participation in regular physical activity.

These findings are similar to findings from other studies, in which dog owners who walked their dogs accumulated physical activity that contributed to meeting recommendations. In an Australian cross-sectional analysis, most dog owners who walked their dogs for more than 1 hour per week were more likely than non-dog owners to meet physical activity guidelines of greater than or equal to 150 minutes of total activity per week (11). In a U.K. prospective study, dog owners significantly increased the number and duration of walks after the first month of dog ownership, and this increase was sustained for the 10 months of the study (12).

Our analysis indicates that among dog walkers, sex and family income make little difference in whether a person walks a dog two or more times in a day or accumulates 30 minutes or more of walking in bouts of at least 10 minutes each. These findings do not, however, suggest whether sex or income makes a difference in participating in dog walking generally. Studies on physical activity participation rates have found that overall walking (15), walking for transportation (16), and leisure-time physical activities (1,16) vary by age, sex, income, and other demographic factors. Because we found that accumulating 30 minutes or more of walking a dog in 1 day is quite common, we urge researchers to find out more about the characteristics of dog walking.

Long-term adherence rates to physical activity programs tend to average only about 50% (7). One reason proposed for low long-term adherence is that typical programs to promote physical activity do not promote purposeful activity (7). The significance of purposeful physical activity in long-term adherence was demonstrated in a report of 10 case studies of individuals who regularly participated in physical activity for 5 to 79 years (7). In seven of the studies, people walked either for transportation or to walk a dog. In three cases, the individuals walked dogs, and these individuals reported walking 3 to 6 miles per day at least 5 days per week for periods ranging from 5 to 15 years. People reported that their regular adherence was due to the need to provide the dog with exercise. The levels of physical activity reported as sustained for years by these people meet or exceed current recommendations.

In addition to providing purposeful activity, dog walking may create a form of social support that has been identified as an effective behavioral strategy for increasing physical activity. The U.S. Task Force on Community Preventive Services strongly recommends social-support interventions in community settings to increase physical activity (17). Included are behavioral strategies such as developing a buddy system and establishing walking groups or other groups. These strategies provide support and motivation for being physically active. Dog walking may support and motivate physical activity by providing companionship and creating expectations in similar ways to human buddy systems. Walking the dog, in contrast to many other forms of physical activity, is relatively easy and convenient to do, because it can generally be done in one's own neighborhood. This is an important consideration, because having convenient exercise settings is a consistent predictor of regular participation in physical activity for many people (18).

Several limitations of this study limit its generalizability. First, although households were randomly selected, the sample was obtained through the responses of participants to open-ended questions on the travel diary; thus, it was not a population-based sample, even though weights were used. Prevalence estimates for dog walking have not been published; however, about 39% of U.S. households have at least one dog (8), and one study suggests that about two thirds of dog owners walk their dogs, and about 90% of them spend more than 10 minutes on their walks (19). We must consider whether certain subgroups of the dog-walking population were underrepresented. People who walk their dog frequently could have disproportionately participated, thereby introducing selection bias that could have resulted in overestimating the time spent in dog walking and reduced the variability in the sample. Second, when the trip purpose was pet care and the travel mode was walking, it was presumed to be dog walking. These trips could have included misclassified trips to the veterinarian or walking other species of pets. The results of this study did not change, however, when we excluded individuals (n = 32) who did not own cars and would therefore likely walk their pets to the veterinarian. Third, the intensity or speed of walking could not be assessed because walking distance and time are subject to recall bias and digit preference. Dog walking of moderate intensity may be less prevalent than this study suggests.

Fourth, for many dog walkers (especially those with certain breeds of dogs or those who often walk in inclement weather), dog walking may be always of short duration (less than 10 minutes) and thus not count at all toward accumulating 30 minutes or more of dog walking in bouts of at least 10 minutes as recommended by the Centers for Disease Control and Prevention and the American College of Sports Medicine (5). Although we can understand the rationale for requiring bouts of at least 10 minutes, we might argue that for dog walkers, short bouts of physical activity are better than sedentary lifestyles. Fifth, proxy interviews for youths aged younger than 16 years may introduce underreporting or overreporting of data.

Finally, the sample design of the NHTS, the low response rate, and the low reporting of dog walking in the diary (n = 1282) might have biased the sample. The 2001 NHTS included a national sample of 25,000 households and oversampled to obtain additional households (N = 37,260) in nine states and transportation regions. Because of the low reporting of dog walking in the open-ended travel diaries, these data may not accurately estimate the characteristics of dog walking and should be interpreted with caution. No differences in characteristics were seen when conducting the analyses with and without sample weights.     

This is the first national study to estimate the contribution of dog walking to physically active lifestyles among people in the United States who walk dogs. The data suggest that dog walking can contribute to a physically active lifestyle. Given the limitations of the study, however, more research is needed to determine the national prevalence of dog walking and its contribution to physically active lifestyles. Because it is purposeful, is convenient for most dog owners, and can regularly motivate and support physical activity, dog walking may address several important barriers to physical activity in humans.

## Figures and Tables

**Table 1:** Selected Characteristics of Dog Walkers in the National Household Travel Survey, United States, 2001–2002

**Characteristic**	**Males, %(n = 526)**	**Females, %(n = 756)**	**Total, %(N = 1282)**

**Age, y**			

<18	9.6	11.3	10.6
18-44	34.6	33.6	34.0
≥45	55.9	55.1	55.4

**Annual family income, $**			

≤50,000	38.7	44.2	41.9
>50,000	61.3	55.8	58.1

**Region**			

Northeast	31.0	30.3	30.6
Midwest	38.0	40.3	39.4
South	17.3	17.3	17.3
West	13.7	12.0	12.7

**Urbanicity**			

Urban	11.6	10.3	10.8
Second city^a^	19.2	23.2	21.5
Suburban	28.0	27.5	27.7
Small town	24.5	23.3	23.8
Rural	16.7	15.7	16.2

a A second city is a secondary population center in an urbanized area.

**Table 2:** Estimated Proportion of U.S. Dog Walkers Who Walked a Dog Two or More Times in 1 Day and Who Walked a Dog at Least Once in 1 Day, 2001–2002

**Characteristic **	**Two or More Dog Walks^a^ in 1 Day% (95% Confidence Interval)**	**One or More Dog Walks^a^ in 1 Day% (95% Confidence Interval)**
**Total**	58.9 (53.3-64.4)	80.2 (75.3–84.4)

**Sex**		

Male	63.4 (56.7-69.7)	78.8 (72.7–83.8)
Female	55.6 (48.5-62.4)	81.3 (74.8–86.4)

**Age, y**		

<18	53.4 (37.7-68.4)	71.8 (56.0-83.5)
18–44	61.0 (52.0-69.3)	78.6 (68.9-85.9)
≥45	57.6 (51.5-63.6)	83.7 (78.1-88.1)
≥18	59.1 (53.5-64.6)	81.4 (76.4-85.5)

**Annual family income, $**		

≤50,000	57.3 (50.0-64.3)	82.8 (76.1-87.8)
>50,000	60.2 (51.8-68.0)	79.1 (71.3-85.2)

**Region**		

Northeast	64.0 (54.3-72.7)	86.2 (77.4-91.9)
Midwest	65.4 (54.9-74.7)	72.3 (62.1-80.6)
South	54.5 (44.2-64.4)	81.0 (70.7-88.3)
West	55.7 (43.3-67.4)	79.9 (68.8-87.7)

**Urbanicity**		

Urban	59.9 (47.6-71.0)	80.9 (69.8-88.6)
Second city^b^	61.0 (47.1-73.3)	84.1 (73.3-91.1)
Suburban	59.6 (50.0-68.5)	78.3 (66.3-86.9)
Small town	58.0 (45.3-69.8)	79.9 (71.6-86.2)
Rural	54.8 (40.1-68.6)	79.3 (67.2-87.8)

a A dog walk was defined as walking a dog for at least 10 minutes.

b A second city is a secondary population center in an urbanized area.

**Table 3:** Estimated Proportions of U.S. Dog Walkers Who Accumulated 30 Minutes or More of Dog Walking in 1 Day From Walks of at Least 10 Minutes, by Selected Characteristics, 2001–2002

**Characteristic**	**% (95% Confidence Interval)**
**Total**	42.3 (37.3-47.5)

**Sex**	

Male	38.3 (31.1-46.0)
Female	45.3 (39.2-51.6)

**Age, y**	

<18	21.7 (12.5-34.8)
18–44	47.4 (38.6-56.4)
≥45	44.3 (38.2-50.6)

**Annual family income, $**	

≤50,000	42.8 (35.1-50.9)
>50,000	42.8 (36.2-49.7)

**Region**	

Northeast	51.7 (41.0-62.2)
Midwest	36.7 (28.8-45.3)
South	43.4 (33.5-53.9)
West	37.6 (27.8-48.5)

**Urbanicity**	

Urban	55.5 (43.3-67.0)
Second city^a^	42.5 (31.7-54.0)
Suburban	37.8 (28.5-48.1)
Small town	40.3 (30.2-51.2)
Rural	42.3 (28.3-57.7)

a A second city is a secondary population center in an urbanized area.

**Table 4:** Adjusted Odds Ratios^a^ of Accumulating 30 Minutes or More of Dog Walking in 1 Day From Walks of at Least 10 Minutes Each, 2001–2002

**Characteristic**	**Odds Ratio (95% Confidence Interval)**

**Sex**	

Male	0.74 (0.49-1.11)
Female	1.00 (ref^b^)

**Age, y**	

<18	0.37 (0.17-0.77)
18–44	1.10 (0.67-1.80)
≥45	1.00 (ref)

**Annual family income, $**	

≤50,000	0.95 (0.61-1.46)
>50,000	1.00 (ref)

**Region**	

Northeast	2.03 (1.08-3.81)
Midwest	1.00 (ref)
South	1.39 (0.77-2.52)
West	1.08 (0.58-2.03)

**Urbanicity**	

Urban	1.77 (0.82-3.79)
Second city^c^	1.18 (0.58-2.39)
Suburban	1.00 (ref)
Small town	1.01 (0.50-2.02)
Rural	1.16 (0.50-2.69)

a Controlling for sex, age, annual family income, region, and urbanicity.

b Ref indicates referent group.

c A second city is a secondary population center in an urbanized area.

## References

[1] (1996). U.S. Department of Health and Human Services. Physical activity and health: a report of the Surgeon General.

[2] Sapkota S, Bowles HR, Ham SA, Kohl HW (2005). Adult participation in recommended levels of physical activity — United States, 2001 and 2003. MMWR Morb Mortal Wkly Rep.

[3] Kruger J, Ham SA, Kohl HW (2005). Trends in leisure-time physical inactivity by age, sex, and race/ethnicity — United States, 1994–2004. MMWR Morb Mortal Wkly Rep.

[4] Grunbaum JA, Kann L, Kinchen S, Ross J, Hawkins J, Lowry R (2004). Youth risk behavior surveillance — United States, 2003. MMWR Surveill Summ.

[5] Pate RR, Pratt M, Blair SN, Haskell WL, Macera CA, Bouchard C (1995). Physical activity and public health. A recommendation from the Centers for Disease Control and Prevention and the American College of Sports Medicine. JAMA.

[6] U.S. Physical Activity Statistics: 1998 U.S. Physical Activity Statistics: Participation in Select Physical Activities [Internet].

[7] Morgan  WP (2001). Prescription of physical activity: a paradigm shift. Quest: The Academy Papers.

[8] U.S. pet ownership statistics [Internet].

[9] Wolfsheimer KJ, Ettigner SJ, Feldman EC (2000). Obesity. Textbook of veterinary internal medicine.

[10] Caring for your dog: the top ten essentials [Internet].

[11] Bauman AE, Russell SJ, Furber SE, Dobson AJ (2001). The epidemiology of dog walking: an unmet need for human and canine health. Med J Aust.

[12] Serpell J (1991). Beneficial effects of pet ownership on some aspects of human health and behaviour. J R Soc Med.

[13] Kushner RF, Jackson D, Baltes A, Shanda-Retelny V, Rudloff K The P-PET study — people and pets exercising together: the role of a companion animal as social support for weight loss and weight maintenance. Paper presented at the North American Association for the Study of Obesity, Annual Scientific Meeting. Paper presented at the North American Association for the Study of Obesity, Annual Scientific Meeting.

[14] (2004). 2001 National Household Travel Survey: User's Guide.

[15] Tudor-Locke C, Ham SA, Macera CA, Ainsworth BE, Kirtland KA, Reis JP (2004). Descriptive epidemiology of pedometer-determined physical activity. Med Sci Sports Exerc.

[16] DATA2010: the Healthy People 2010 database.

[17] (2001). Increasing physical activity. A report on recommendations of the Task Force on Community Preventive Services. MMWR Recomm Rep.

[18] Dishman RK, Sallis JF, Orenstein DR (1985). The determinants of physical activity and exercise. Public Health Rep.

[19] Slater MR, Robinson LE, Zoran DL, Wallace KA, Scarlett JM (1995). Diet and exercise patterns in pet dogs. J Am Vet Med Assoc.

